# Impact of Diabetes Mellitus in Patients with Pancreatic Neuro-Endocrine Tumors: Causes, Consequences, and Future Perspectives

**DOI:** 10.3390/metabo12111103

**Published:** 2022-11-11

**Authors:** Lorena Hernandez-Rienda, Maria Isabel del Olmo-García, Juan Francisco Merino-Torres

**Affiliations:** 1 Endocrinology and Nutrition Department, University and Politecnic Hospital La Fe, 46026 Valencia, Spain; 2 Joint Research Unit on Endocrinology, Nutrition and Clinical Dietetics, Health Research Institute Hospital La Fe-University of Valencia, 46026 Valencia, Spain; 3 Department of Medicine, Faculty of Medicine, University of Valencia, 46010 Valencia, Spain

**Keywords:** neuroendocrine tumors, diabetes mellitus, type 3 diabetes, metformin

## Abstract

Diabetes mellitus (DM) and pancreatic neuroendocrine tumors (pNETs) are two entities closely linked together. DM has been described as a risk factor for the development of pNETs and for the aggressiveness of the disease. On the other hand, DM due to pNETs is frequently undiagnosed or misclassified as type 2 DM when it is due to type 3 DM. In addition, metformin, a commonly prescribed drug for type 2 DM, has an antiproliferative property and is gaining increasing attention as an antitumor agent. This review article presents the findings published in the last few years on pNETs and DMs. Emphasis will be placed on DM as a risk factor, pNET as a risk factor for the development of type 3 DM, the management of type 3 DM on pNET, and DM as a prognostic factor in patients with pNET, as well as the future clinical implications of DM in these patients. The coexistence of DM and pNET is extensively presented. It is important to perform future clinical trials, which are necessary to establish the role of metformin on pNET disease. Increasing awareness among professionals managing pNET on the importance of a correct DM diagnosis and management of the disease must be a priority due to the implications on mortality and comorbidities it may have in these patients.

## 1. Introduction

Neuroendocrine tumors (NETs) are tumors that arise from neuroendocrine cells, which are widely distributed throughout the body and therefore can appear in different organs such as the foregut, midgut, hindgut, pancreas, lung, or other unusual locations. The pancreatic gland is one of the most commonly affected organs, accounting for 1–2% of the totality of tumors in the pancreas [1]. Likewise, pNETs represent 4–7% of the total gastroenteropancreatic NETs [2]. The incidence of NETs has been rising over time, probably due to the improvement of imaging techniques [3,4].

In order to ensure normal body function, tight control of blood glucose levels is necessary. The pancreas represents a key player in glucose control by secreting insulin (beta cells) and its antagonist glucagon (alpha cells) [5]. Therefore, associations between diabetes mellitus (DM) and pancreatic NETs (pNET) are a fact.

DM has been described, along with smoking, as a probable risk factor for the development of pNET [6]. The incidence DM reported is high in patients with pNET [7]. These patients may have functioning tumors that interfere with glucose metabolism, such as glucagonoma, somatostatinoma, GH secretion, or ectopic ACTH secretion. In addition, tumor size may alter insulin secretion by inducing atrophy or destruction of the pancreas [8]. On the other hand, multiple treatments that interfere with glucose metabolism, such as extensive pancreatic surgeries, systemic treatments with somatostatin analogues, mTOR inhibitors, or chemotherapy, may cause or worsen a pre-existing DM [9]. Moreover, management of these DMs may be complicated, added to the fact that DM has been described as a risk factor for prognosis while certain treatments for DM, such as metformin, may improve survival [9].

This review article presents the findings published in the last few years on pNETs and diabetes. Emphasis will be placed on describing how type 2 DM has been described as a strong risk factor for the development of pNET, as well as how pNET and its management are a risk factor for the development of type 3 DM. On the other hand, recommendations on management and diagnosis of type 3 DM in pNET will be addressed. Finally, comments will be made on DM as a prognostic factor in patients with pNET as well as the future clinical implications of DM and its treatments in these patients.

The search strategy was designed to find studies and reviews including a combination of Medical Subject Headings (MeSH) and non-MeSH keywords related to diabetes and pNETs: “type 3 diabetes mellitus” or “diabetes mellitus” and “pancreatic neuroendocrine tumors”.

## 2. Is Diabetes Mellitus a Risk Factor for the Development of pNETS?

Risk factors for the development of pNETS are not well understood; several systematic reviews and meta-analyses have been published that explore type 2 DM as a risk factor for the development of pNETs.

A meta-analysis and systematic review published in 2015 explored different risk factors such as pre-existing DM, smoking, first-degree relatives with cancer, or alcohol on the development of pNET. Three different cohorts were studied, and the pooled estimated OR for DM was 2.74, showing that, for the study population, pre-existing DM exposure was a strong risk factor for the development of pNET (95% CI 1.63–4.62, *p* < 0.01). In this study, only a first-degree relative with cancer and DM were risk factors for the development of pNET, with DM being the strongest risk factor. Of the three cohorts considered in this study, only one accounted for the role of recent-onset diabetes, for which the association appeared to be stronger [6,10,11,12].

A similar meta-analysis, which considered the same 3 cohorts, provided an OR estimate of 2.76 for pre-existing DM as a risk factor for the development of pNETS [13]. This same group analyzed the effect of body mass index (BMI) as a predictive factor, and an adjusted effect estimate of 1.37 was obtained (95% CI 0.25–7.69, *p* < 0.001) for individuals with obesity compared to normal-weight individuals and the development of pNETs [11,12,13,14].

A study that explored the association between a large number of potential risk or protective factors for the development of sporadic pNETs was performed as a multicenter European study and published in 2017 [15]. In this study, recent DM was defined as the DM that was diagnosed 12 months prior to the pNET diagnosis, and non-recent DM as being diagnosed >12 months. An increased risk of pNET occurrence with non-recent DM was reported with an OR of 1.89 (95% CI 1.17–3.05, *p* = 0.008). Non-recent onset DM resulted in a consistent risk factor for intervals within 1 and up to 5 years from diagnosis; however, in those with a DM onset of more than 5 years, the resulting association was not significant.

The molecular mechanisms linking DM to pNET development are largely unknown. On the one hand, some authors point out that DM may be an early paraneoplastic condition or a tumor-induced impairment rather than a real promotion of cancer initiation [8,16]. Alternatively, an explanation for this association would be that beta cells typically express low levels of antioxidant enzymes, and therefore a relationship between oxidative stress and DM could potentiate somatic mutations that would trigger the development of sporadic pNETs. This way, some authors support the idea that rather than a sign of the disease, long-standing diabetes is a risk factor for the development of pNETs [15].

## 3. What Is the Prevalence and Physiopathology of Diabetes in Patients with pNETs?

Data which reports the prevalence of DM in pNETs is scarce. Two studies in the Chinese population have described a prevalence of DM between 20.2 and 16.9%, respectively [7,17].

The incidence of DM in the pNET patient population has been studied recently by another Chinese group, excluding insulinomas. In these series, the prevalence of DM was higher, at 26% in patients ≥ 60 years old and 12.1% in patients < 60 years old [18].

Diabetes may be diagnosed based on plasma glucose criteria, either the fasting plasma glucose (FPG) value or the 2-h plasma glucose (2-h PG) value during a 75-g oral glucose tolerance test (OGTT) or HbA_1C_ criteria. The DM classification is complex and not limited to the classical types 1 and 2 DM. Hence, the American Diabetes Association (ADA) differentiates five types of diabetes based on the pathophysiology: type 1 (β-cell destruction leading to an absolute insulin deficit), type 2 (with insulin resistance and a relative insulin secretion deficit), type 3 (other specific types), type 4 (other genetic syndromes occasionally associated with diabetes), and type 5 (gestational DM). Moreover, DM type 3 is subdivided into 3A (genetic defects of β cell function), 3B (genetic defects of insulin action), 3C (exocrine pancreas disease), 3D (endocrinopathies), 3E (drugs or chemical agents), 3F (infections), and 3G (uncommon forms of autoimmune diabetes) [19].

**Figure 1:**
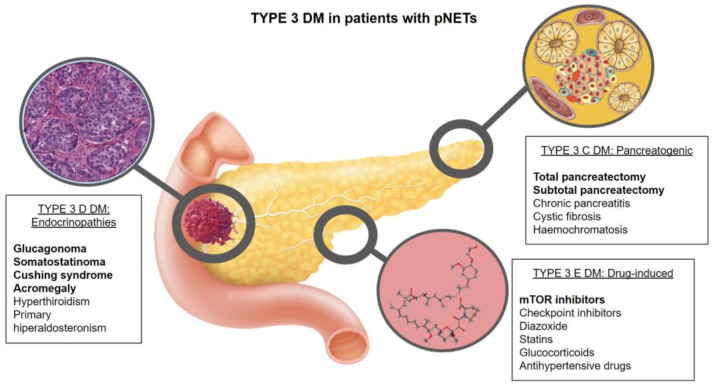
Schematic figure of the pathophysiology of type 3 DM in patients with pNETs.

The most characteristic types of DM due to pNETs are types 3C, 3D, and 3E, although obviously, patients with pNETs can have preexisting or coexisting type 2 or type 1 DM (Figure 1).

### 3.1. Type 3C DM (Exocrine Pancreas Disease or Pancreatogenic Diabetes) (T3CDM)

There is insufficient knowledge that differentiates T2DM and T3CDM, frequently leading to an inadequate DM diagnosis and management, along with the potential entailing nutritional and metabolic implications. Damage to the pancreatic gland, that is to say, endocrine and exocrine function, disrupts the complex interplay between nutrient digestion and absorption, as well as causing a deficiency of incretin hormones, such as PP, insulin, and glucagon [20]. Some degree of exocrine dysfunction and malabsorption and/or maldigestion of nutrients coexist. T3CDM is typically a secondary diabetes due to the destruction or loss of pancreatic tissue, resulting in a clinically relevant condition that represents 8% of the totality of all diabetic subjects in Western populations. Within this type of DM, different pathological entities are included: chronic pancreatitis (76%), pancreatic neoplasia (9%), hemochromatosis (8%), cystic fibrosis (4%) and pancreatic resection (3%) [21,22]. All these etiologies cause either inflammation and/or fibrosis of the pancreas, which damages both exocrine and endocrine functions.

Since it is not always easy to diagnose and classify a patient with T3CDM correctly, proposed diagnostic criteria are shown in Table 1 [23,24].

**Figure 2:**
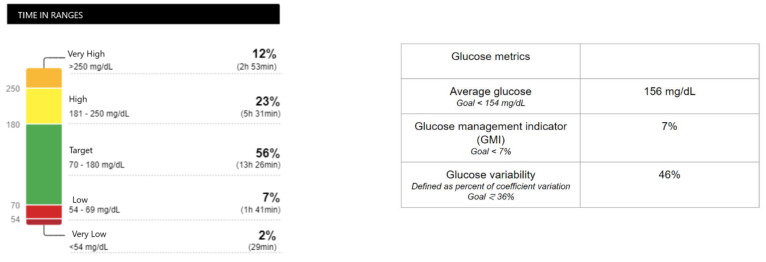

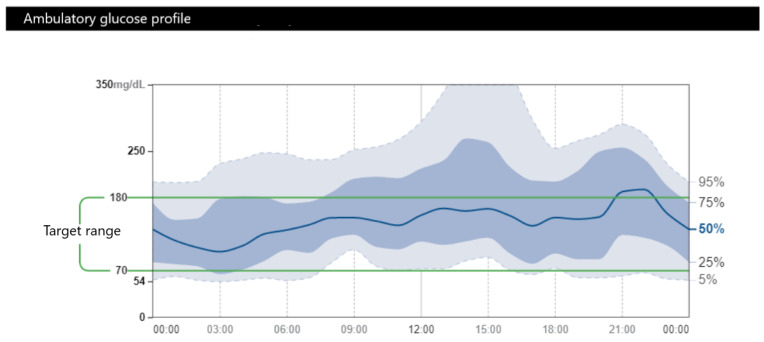
Ambulatory glucose profile (AGP) report of continuous glucose monitoring (CGMS) in a patient with total pancreatectomy due to an 8 × 6 × 7.5 cm pNET.

pNET-T3CDM is usually secondary to surgical procedures on the pancreas. pNET patients are frequently treated with pancreatic resection. Pancreatic resection carries a risk of pancreatic endocrine insufficiency, particularly in total pancreatectomies, in which there is an absolute insulin deficiency and a need for lifelong insulin replacement. Due to the paradoxical combination of normal or increased peripheral insulin sensitivity and decreased hepatic insulin sensitivity, clinical management may be challenging in these circumstances. Patients have high glycemic variability because their hepatic glucose production is not suppressed. They easily become hyperglycemic and also experience severe hypoglycemic episodes when insulin replacement is not excessive (Figure 2).

A recent systematic review examined the incidence and risk factors for the development of DM following partial pancreatic resection. The data on the prevalence of T3CDM in partial pancreatomies is limited. Information on T3CDM by type of resection showed a considerable difference in new-onset DM depending on the surgical procedure. Distal pancreatectomy resulted in the highest incidence of new-onset DM (21%), followed by pancreaticoduodenectomy (16%) while central pancreatectomy presented the lowest incidence of DM (6%). In one study, the median time to develop diabetes was up to 15 months, indicating the need for ongoing screening for T3CDM. Higher preoperative HbA1C (>5.7%), lower remnant pancreatic volume, and fasting plasma glucose were the risk factors with the strongest associations with the onset of T3CDM [25].

Another study [26] looked at how different pancreatic diseases (pancreatic cancer, pancreatitis, and benign tumors of the pancreas) affected glucose homeostasis and insulin secretion after a 50% partial pancreatectomy. While glucose concentrations can paradoxically improve after pancreatic head resection, the authors found that insulin secretion decreased following tail and head pancreatic resection. Obesity and glucose levels prior to surgery were risk factors for DM and hyperglycemia following pancreatic surgery. Other groups appear to agree that, in addition to beta cell reduction, the worsening of glucose control following surgery may be caused by uncontrolled or undiagnosed diabetes mellitus prior to surgery. On the other hand, as previously commented, in some cases they observed an improvement in glycemic control after pancreatic cancer resection which, they attribute to insulin resistance caused by the tumor itself or even to the weight loss induced by the failure of exocrine function, which could increase peripheral insulin sensitivity [27]. However, only large pancreatectomies can determine malabsorption due to diminished pancreatic exocrine function, which is generally well controlled by the rapid introduction of replacement therapy with pancreatic enzymes; thus, the improvement in glycemic control observed in certain patients after pancreatectomy shows contradictory results.

### 3.2. Type 3D DM (Endocrinopathies) (T3DDM)

Non-functional pNETs are the most common, representing between 60–90% of the most recent series. Within functional pNETs, insulinoma and gastrinoma are the most frequent [28]. However, several functioning pNETs may cause, due to hormonal secretion, this type 3D DM.

#### 3.2.1. Glucagonoma

Glucagonomas are an extremely rare NETs that have been described as having an incidence of 0.01–0.1 new cases/106 population/year. The total of all glucagonomas have been found on the pancreatic gland, with a malignant potential between 50–80% and an association with type 1 multiple endocrine neoplasia (MEN1) between 1–20% [28]. Glucagon increases hepatic glucose output by increasing gluconeogenesis and glycogenolysis, leading to an increase in plasma glucose levels and therefore to DM. The typical syndrome that is frequently brought on by glucagonomas includes: a rash known as necrolytic migratory erythema, painful glossitis, cheilitis, and angular stomatitis, normochromic normocytic anemia, deep vein thrombosis, weight loss, mild DM, hypoaminoacidemia, low zinc levels, and depression.

DM due to a glucagonoma is usually mild and occurs in 68–80% of patients. In the largest study group reported, although only 38% of subjects had overt DM at presentation, eventually 76% of the patients developed DM, 75% of whom required insulin therapy [29]. Removal of the tumor, if possible, results in remission of the diabetes [30].

In glucagonomas, glucose tolerance may be related to tumor size, typically when it is greater than 4–5 cm. Patients with higher burden liver disease tend to have higher fasting plasma glucagon levels than patients with localized disease [8,31].

#### 3.2.2. Somatostatinoma

Somatostatinomas are also extremely rare tumors that may present with a triad of diarrhea/steatorrhea, DM, and gallstones. Somatostatinomas may appear in the pancreatic gland (55%), but also on the gastrointestinal tract, usually the duodenum or jejunum (44%). Malignant potential is described in >70% of the cases, and there is an association with MEN1 in 45% of the cases [28]. Somatostatin inhibits insulin secretion, which can result in elevations in plasma glucose levels; 75% of somatostatinomas located on the pancreatic gland will develop DM, while those in the gastrointestinal tract will only develop DM in 10% of the cases [30]. DM, like in glucagonomas, is relatively mild; in this case, the absence of ketoacidosis is thought to be caused by the simultaneous suppression of glucagon and insulin secretion by somatostatin [8]. Removal of the tumor will result in a DM remission.

#### 3.2.3. Other Ectopic Hormone Secretion

Other, more unfrequent pNETs that secrete ACTH, causing ectopic Cushing disease, or GRH, causing acromegaly, can also cause DM due to the hormonal syndrome.

Pancreatic ACTHoma accounts for between 4 and 16 percent of all ectopic Cushing’s syndrome cases. DM is present in 20–47% of patients with Cushing’s syndrome [32]. DM prevalence varies based on Cushing’s syndrome etiology: 74% are ectopic, 34% are adrenal, and 33% are pituitary. Glucocorticoids disrupt glucose metabolism primarily by stimulating hepatic gluconeogenesis and inducing insulin resistance in the liver and muscles. Glucose abnormalities are usually detected by high postprandial glucose and HbA1c, while fasting glucose may be normal. A multicenter retrospective analysis of ectopic Cushing’s syndrome revealed that pNETs were the source of ectopic secretion of CRH/ACTH in 15.5% of cases [33]. Treatment of ectopic Cushing’s syndrome by the removal of the ectopic ACTH tumor results in a marked improvement of glucose metabolism and a remission of DM. However, in patients with persistent Cushing syndrome, drug therapy may be necessary to restore cortisol levels and glucose abnormalities [33].

Growth hormone-releasing hormone syndromes usually involve the lungs but can also be located in the pancreatic gland in 30% of cases. In patients with acromegaly, insulin resistance is the major abnormality leading to disturbances in glucose metabolism. Treatment is directed to the cause of the acromegaly; in this case, successful removal of the GRH-secreting pNET improves hyperglycemia, and glucose metabolism has been reported to normalize in 23–58% of people with preoperative diabetes after surgical cure of acromegaly [28].

### 3.3. Type 3E DM

A large number of different drugs have been shown to adversely affect glucose homeostasis. Some of the treatments that are used on advanced pNETS may induce pharmacological DM. Glucose abnormalities are classified according to the U.S. Department of Health and Human Services in five grades of severity (Table 2) [34].

#### 3.3.1. Somatostatin Analogues (SSA)

SSAs, which are commonly used as first-line therapy in pNETs due to their antisecretory and antiproliferative properties, may cause glucose abnormalities due to their inhibitory effects on pancreatic endocrine secretion. Somatostatin inhibits insulin secretion by the beta cell through SSTR2 and SSTR5 receptors [35]. By acting predominantly on the SSTR2, first-generation SSAs such as octreotide and lanreotide have a modest impact on glucose metabolism. In fact, no glucose alterations were described on the PROMID trial [36], and on the CLARINET study, hyperglycemia was described in 5% of the treatment patients compared to none in the placebo group [37]. However, pasireotide has an effect on STTR2 and STTR5, which leads to a higher incidence of glucose abnormalities. In the phase III clinical trial that compared pasireotide LAR with octreotide LAR in refractory carcinoid syndrome patients, pasireotide showed a significantly higher rate of hyperglycemia (28.3% vs. 5.3%) together with a higher incidence of grade 3–4 hyperglycemia with two patients discontinuing treatment due to grade 4 drug-related hyperglycemia [38]. Glucose abnormalities due to SSA are generally mild and dose-dependent, can be managed with antidiabetic therapy if necessary, and tend to resolve within the first few weeks of treatment.

#### 3.3.2. Everolimus

The RADIANT-3 trial was a randomized, double-blind, placebo-controlled phase III trial that demonstrated significant clinical benefit of everolimus in the largest number of patients with advanced pancreatic NETs (panNET) [39]. Based on this result, the Food and Drug Administration (FDA) approved everolimus as an anti-tumoral drug for the treatment of panNETs with progressive disease. Insulin resistance due to a reduction of the post-receptor insulin signaling pathway and the reduction of insulin secretion through a direct effect on the pancreatic beta cells, as well as the reduction of glucose transport and intracellular use in peripheral tissues, are the primary causes of the adverse effects of mTOR (mammalian target of rapamycin) inhibitors on glucose metabolism [40].

When used as an anticancer drug, mTOR inhibitors significantly increase the risk of new-onset diabetes and induce a 5-fold increase in the risk of severe hyperglycemia. The risk increase is suggested to be caused by dose increases, and the estimated risk is between 12–50% of any grade of severity. In addition, the risk of hyperglycemia attributable to everolimus varies by tumor type [41,42]. Among prospective trials performed with everolimus in pNET patients, the RADIANT-1 described 13% of hyperglycemia (4% grade 3/4) [43], and a similar 13% incidence of hyperglycemia (5% grade 3/4) in the RADIANT-3 trial [39]. In the COOPERATE-2 trial, which evaluated everolimus +/− pasireotide, a higher incidence of hyperglycemia was observed when everolimus was combined with pasireotide (76% versus 27%), with an incidence of grade 3/4 of 37% versus 11% [44].

When compared to controls, meta-analyses have demonstrated that rates of hyperglycemia, hypercholesterolemia, and hypertriglyceridemia were significantly higher. In a few instances, the consequences were severe enough to set off diabetic ketoacidosis [42].

#### 3.3.3. Interferon Alpha

Interpheron alpha may cause DM, but in this case, unlike the previously commented drugs, it would not be type 3E DM. IFN-α through immune activation and direct beta-cell toxicity mechanisms, may induce autoimmune type 1 DM, resulting in permanent insulin therapy dependency [45]

#### 3.3.4. Cytotoxic Chemotherapy

Streptozocin, a systemic chemotherapy agent approved for high-grade pNETs, is cytotoxic to pancreatic beta cells. Streptozotocin accumulates in pancreatic beta cells via the GLUT2 glucose transporter. Hydroxyl radicals are produced and are ultimately responsible for the death of the beta cells, which have a particularly low antioxidative defense capacity [46].

## 4. Glycemic Goals and Management of DM in Patients with pNETs

Glycemic targets in pNET patients should be guided by ADA and European Association for the Study of Diabetes (EASD) recommendations. Glycemic goals should be assessed based on a personalized approximation, taking into consideration the following risks potentially associated with hypoglycemia and other drug adverse events, disease duration, life expectancy, important comorbidities, established vascular complications, patient preference and resources, and a support system. Clinicians should aim to achieve an HbA_1c_ level between 7 and 8% in most patients with DM. However, clinicians should treat patients with DM to minimize symptoms related to hyperglycemia and avoid targeting an HbA_1C_ level in patients with a life expectancy less than 10 years due to advanced age (80 years or older) or chronic conditions because the harms outweigh the benefits in this population. Therefore, pNET patients’ goals will be different in those with localized disease, in which HbA_1C_ should be aimed <7%, than in those with limited survival, in which goals could be between 7–8% or even avoid targeting glucose control in those with a low life expectancy [19].

**Figure 3:**
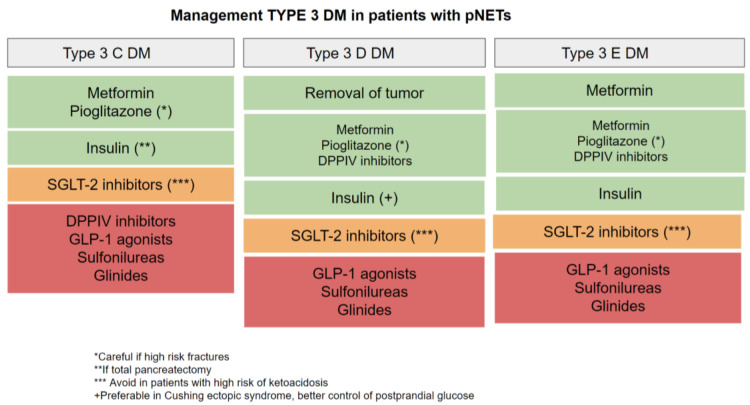
Pharmacological treatment of diabetes in patients with pNETs.

A therapeutic approach should take into account different aspects such as the underlying causes responsible for glucose imbalance (pre-existent T2DM, T3CDM, T3DDM, and T3EDM), pancreatic reserve (especially after resection), liver and kidney function, or the presence of nutritional imbalances. Data about diabetes therapy for patients with pNETs are still lacking, and recommended therapies are generally based on the current ADA and EASD treatments for type 2DM, however, there are certain peculiarities that should be taken into account (Figure 3).

### 4.1. Type 3C DM

T3CDM poses a unique management challenge in comparison to Type 2 DM due to the nature of glucose homeostasis since it is a patient with reduced pancreatic function. Treatment includes both management of hyperglycemia and exocrine pancreatic insufficiency. The aim of treatment is not only to control hypoglycemia and hyperglycemia but also malabsorption, malnutrition, and chronic DM complications.

General recommendations for the patient include: avoiding skipping meals and eating small quantities frequently, including complex carbohydrates and avoiding simple sugars; monitoring glucose levels with capillary blood glucose or a continuous glucose sensor; ensuring pancreatic enzyme replacement therapy; nutritionist support; and avoiding alcohol and tobacco.

Regarding treatment for glucose control, metformin seems to be the drug of choice (as in type 2 diabetes), which also seems to act as a protective factor against the development of pancreatic cancer, although its main disadvantage is intolerance or worsening of the digestive symptoms. If control is not optimal with metformin monotherapy, the addition of a second drug could be considered, opting for pioglitazone due to its increased sensitivity to insulin; however, as the risk of fracture increases, it must be used with caution. Sulfonylureas and glinides are usually avoided due to the risk of hypoglycemia, as are incretin-based therapies (DPP4 inhibitors and GLP-1 analogs) due to underlying pancreatic disease. However, studies that evaluate the risk of pancreatitis in patients with T3CDM treated with incretins are lacking. As long as the patient is not susceptible to diabetic ketoacidosis, the recently introduced sodium-glucose co-transporter-2 (SGLT2) inhibitors may be used in patients both with insulin resistance and those with altered insulin secretion [8,19].

In advanced phases with a predominance of insulin secretion deficit, total pancreatectomy, intolerance or contraindication of the previous drugs, or a lack of achievement of control objectives, insulin would be the treatment of choice. Due to the increased risk of hypoglycemia in these patients when they are on intensive insulin therapy (basal-bolus regimen), a continuous interstitial glucose monitoring system should be considered [19,47,48,49,50].

### 4.2. Type 3D DM

DM due to functional tumors such as glucagonomas and somatostatinomas is relatively mild and resolves with the removal of the tumor. It can usually be controlled with diet, oral hypoglycemic agents, or small doses of insulin. On the other hand, in cases of metastatic pNETs, systemic treatment will treat hormonal secretion, therefore controlling impaired glucose secretion.

In the case of functional tumors, where hormonal hypersecretion of counterregulatory hormones induces insulin resistance, treatment with insulin sensitizers such as metformin and pioglitazone may be preferred. However, pioglitazone in patients with Cushing’s disease may increase bone loss and fracture risk and therefore has to be used with caution. Since Cushing’s syndrome is characterized by elevated postprandial glucose levels, medications that lower these levels—such as DPPIV inhibitors, GLP1 receptor agonists, and rapid-acting insulin—may prove to be very beneficial. In glucocorticoid-treated patients requiring a basal-bolus insulin regimen, which is more frequent in ectopic ACTH secretion of pNETs, a higher dose requirement of short-acting insulin compared to basal insulin is frequently required. Patients with Cushing’s syndrome frequently require higher insulin doses to maintain glycemic control due to insulin resistance [30].

The use of DPPIV inhibitors and GLP-1 receptor agonists in these pNETs is somehow controversial, and there is scarce data in the literature about their safety. It seems that DPPIV inhibitors may have an anti-inflammatory effect on certain pNET tumors [51], while GLP-1 agonists are generally considered unsuitable due to their potential proliferative properties [52].

### 4.3. Type 3E DM

Clinicians should be aware of the adverse effects of hyperglycemia especially when using SSAs, especially pasireotide and/or everolimus to treat pNETs. These patients should be monitored for impaired glucose regulation before treatment, and glucose monitoring, which includes fasting glucose and HbA1c levels, should be performed during the follow-up. In those patients with preexisting DM or with risk factors, severe hyperglycemia is more likely to be develop and therefore glucose monitoring should be performed more frequently [8].

Appropriate dietary and lifestyle measures represent the first-choice treatment of such drug-induced hyperglycemia adverse events. Pharmacological treatment in this type of DM should take into consideration that all of these patients have advanced disease, target goals should be personalized, and complications may be more frequent. Metformin should be the first-choice treatment in these patients due to its antiproliferative property (commented posteriorly). As second-step treatment, pioglitazone may be considered as well as DPPIV inhibitors (due to anti-inflammatory properties) or even SGLT-2 inhibitors. GLP-1 agonists should be avoided due to their possible proliferative properties, and in the case of off-target glucose control, insulin treatment should be initiated [8,19].

## 5. Impact of Neuroendocrine Tumors on the Survival, Tumor Stage, and Tumoral Growth in Patients with Diabetes

DM2 has not only been described as a risk factor for the development of pNETs but has also been postulated to influence other aspects of the established disease, such as the location of the pNET, invasion, or tumoral stage. Fan et al. described that DM2 patients presented a higher proportion of non-functional pNETs, a higher proportion of tumors located in the body and tail of the pancreas, and more advanced disease when they were older than 60 years. On the other hand, patients with pNET had a higher incidence of nerve invasion and a higher proportion of G3 tumors. The proportion of DM2 patients with metastases was also 2-fold higher in this group of patients. However, DM2 was not an independent predictor of overall survival or a factor in prognosis [7]. Other investigators conclude that non-recent onset diabetes was associated with a more advanced stage at diagnosis (TNM III–IV vs. TNM I–II, respectively, 23.3% vs. 11.8%, *p*  =  0.05) and with a G3 vs. G1–2 tumor (40.9% vs. 14.9%, *p*  =  0.006, respectively) [15]. An Italian group reported a greater tumor size in patients with DM [53].

On the other hand, metformin, a commonly prescribed drug for type 2 DM, has an antiproliferative property and is gaining increasing attention as an antitumor agent [54]. This protective effect of metformin on cancer has been published and suggested as a decreased risk for pancreatic, colon, lung, breast, and liver cancer. In terms of cell signaling, metformin stimulates AMP-activated protein kinase (AMPK), which reduces glycogenolysisis and hepatic glucoeneogenesis and increases glucose uptake by the muscle. AMPK activation also suppresses mTORC1 which is a key regulator of the proliferation of cancer cells. AMPK-metformin activation may also result in cell cycle arrest or apoptosis [55].

For the first time, a significant association between metformin and a longer progression-free survival (PFS) in patients with advanced pNETS was found in a small retrospective study of 31 patients with advanced grade pNETs who were treated between 2009 and 2012. In this study, all of the patients were treated with octreotide LAR and everolimus until disease progression. DM patients were treated with insulin and metformin; this way, DM patients presented a PFS of 29 months compared to 11 months in non-DM patients (*p* = 0.018). When DM patients were subdivided on the basis of their antidiabetic treatment, PFS in patients treated with metformin was twice as long as that of subjects on insulin (36 vs. 17 months), suggesting that metformin [56]. This same group of investigators previously conducted a larger, retrospective multicentric analysis with 445 patients and advanced panNETs.

The primary objective of the study was to assess the potential association between T2DM and PFS in advanced pNET patients. The secondary objective was to study the relationship between metformin treatment and diabetic patients’ clinical outcomes (PFS and OS). Everolimus, with or without somatostatin analogues (SSAs), was administered to participants in this study, and they were divided according to the onset of DM—before or during treatment. DM patients had a significantly longer PFS than non-DM patients (median 32.0 versus 15.1 months), and metformin-treated patients had a significantly longer PFS than patients on other glucose-lowering medications (median: 44.2 compared to 20.8 months). Metformin’s beneficial effect on PFS was unaffected by metformin dosage, glycemic status, or concurrent antitumor treatment. According to additional evidence, the study demonstrated longer PFS in patients who were on metformin treatment before or within 3-months of everolimus or SSA initiation when compared with both patients who did not take metformin and patients who started metformin more than 3 months after treatment initiation [9].

The CLARINET study post hoc analysis, which looked at how DM and metformin use affected GEPNET, has recently been published. A median PFS was not reached independently of DM status in lanreotide-treated patients. Based on DM status, there were no significant differences in PFS (HR = 0.82, 95% CI 0.39–1.73; *p* = 0.848). However, in the placebo arm, there was a tendency towards lower PFS in patients with DM when compared with those without DM (median PFS: 60.0 weeks and 72.1 weeks (HR = 1.82, 95% CI 1.05–3.15; *p* = 0.052). Patients in the placebo group taking metformin had a significantly (97%) longer median PFS when compared with patients not receiving concomitant metformin: 97.7 weeks compared with 50.7 weeks (HR = 0.03, 95% CI 0.00–0.63; *p* = 0.009) [57].

## 6. Conclusions and Future Perspectives

As a matter of fact, DM and pNETs are two entities closely linked together. The incidence of DM is high in patients with pNETs and a causal relationship between the two entities has been described. Particularly, pNETs patients are prone to developing a distinct type of DM, type 3 DM, which is often misdiagnosed as T2DM due to insufficient knowledge. An adequate T3DM diagnosis has to be made in these patients due to the impact of its management not only on metabolic status, but also on nutritional status and prognostic factors. However, data about DM therapy for patients with pNETs is still lacking, and prospective studies are necessary in order to recommend certain treatments over others.

On the other hand, despite promising data from retrospective and post-hoc analyses, prospective studies are needed to assess whether metformin can really improve clinical outcomes in patients with advanced pNETs, not only in DM patients, but also in those with a normoglycemic status when used in combination with standard systemic treatments.

To date (October 19 2022), there are two prospective treatments evaluating the use of metformin in NET patients. The MetNET1 study, a phase II study, will investigate the antiproliferative potential of metformin in combination with everolimus and octreotide in well-differentiated pNET [58]. The main aim of this study is to evaluate the PFS rate after 12 months of treatment. The secondary objectives are safety, overall survival, and response rate evaluation. Also, a sub-study analysis will be performed, which will evaluate the levels of circulant biomarkers (IL-6, IGF-1) in blood samples.

The MetNET2 study, a pilot, one-arm, open-label, prospective study to evaluate the safety of Lanreotide 120 mg in combination with Metformin in patients with advanced, progressive GI, or lung carcinoids [59]. The aim of this study is to verify the safety of a concomitant administration of Lanreotide 120 mg with Metformin in advanced, progressing gastro-intestinal or lung carcinoid patients, by accurately monitoring patients from a tolerability point of view throughout the entire study.

## Figures and Tables

**Table 1:** Diagnostic criteria for T3CDM.

Major Criteria (All Must Be Fulfilled)	Minor Criteria
Exocrine pancreatic insufficiency	Absence of pancreatic polypeptide secretion
Alteration of the anatomical appearance of the pancreas as evidenced by imaging studies (endoscopy, ultrasound, MRI, or scanner)	Decreased insulin secretion
Absence of anti-beta cell antibodies (positive in type 1 diabetes/LADA)	Low levels of fat-soluble vitamins (A, D, E, K)
	Nutrient malabsorption requiring pancreatic enzyme supplementation

**Table 2:** Common Terminology Criteria for Adverse Events: glucose intolerance and hyperglycemia.

Severity Grade	Glucose Intolerance	Hyperglycemia
G1	Asymptomatic; clinical or diagnostic observations only; intervention not indicated	Abnormal glucose above baseline with no medical intervention
G2	Symptomatic; dietary modification or oral agent indicated	Change in daily management from baseline for a diabetic; oral antiglycemic agent initiated; workup for diabetes
G3	Severe symptoms; insulin indicated	Insulin therapy initiated; hospitalization indicated
G4	Life-threatening consequences; urgent intervention indicated	Life-threatening consequences; urgent intervention indicated
G5	Death	Death
